# A comparison of gene transcription profiles of domesticated and wild Atlantic salmon (*Salmo salar* L.) at early life stages, reared under controlled conditions

**DOI:** 10.1186/1471-2164-15-884

**Published:** 2014-10-09

**Authors:** Beatrix Bicskei, James E Bron, Kevin A Glover, John B Taggart

**Affiliations:** Institute of Aquaculture, School of Natural Sciences, University of Stirling, Stirling, FK9 4LA UK; Institute of Marine Research, Population genetics, PO Box 1870, N-5817 Bergen, Norway

**Keywords:** Domestication selection, Microarray, Atlantic salmon, Gene expression, Farm escapees, Maternal effects

## Abstract

**Background:**

Atlantic salmon have been subject to domestication for approximately ten generations, beginning in the early 1970s. This process of artificial selection will have created various genetic differences between wild and farmed stocks. Each year, hundreds of thousands of farmed fish escape into the wild. These escapees may interbreed with wild conspecifics raising concerns for both the fish-farming industry and fisheries managers. Thus, a better understanding of the interactions between domesticated and wild salmon is essential to the continued sustainability of the aquaculture industry and to the maintenance of healthy wild stocks.

**Results:**

We compared the transcriptomes of a wild Norwegian Atlantic salmon population (Figgjo) and a Norwegian farmed strain (Mowi) at two life stages: yolk sac fry and post first-feeding fry. The analysis employed 44 k oligo-microarrays to analyse gene expression of 36 farmed, wild and hybrid (farmed dam x wild sire) individuals reared under identical hatchery conditions. Although some of the transcriptional differences detected overlapped between sampling points, our results highlighted the importance of studying various life stages. Compared to the wild population, the Mowi strain displayed up-regulation in mRNA translation-related and down regulation in nervous and immune system -related pathways in the sac fry, whereas up-regulation of digestive and endocrine activities, carbohydrate, energy, amino acid and lipid metabolism and down-regulation of environmental information processing and immune system pathways were evident in the feeding fry. Differentially regulated pathways that were common among life stages generally belonged to environmental information processing and immune system functional groups. In addition, we found indications of strong maternal effects, reinforcing the importance of including reciprocal hybrids in the analysis.

**Conclusions:**

In agreement with previous studies we showed that domestication has caused changes in the transcriptome of wild Atlantic salmon and that many of the affected pathways are life-stage specific We highlighted the importance of reciprocal hybrids to the deconvolution of maternal/paternal effects and our data support the view that the genetic architecture of the strains studied highly influences the genes differentially expressed between wild and domesticated fish.

**Electronic supplementary material:**

The online version of this article (doi:10.1186/1471-2164-15-884) contains supplementary material, which is available to authorized users.

## Background

Commercial Atlantic salmon (*Salmo salar* L.) aquaculture was first initiated in Norway during the late 1960s, and has grown rapidly to become one of the most economically significant global aquaculture industries (FAO 2013). Current world-production is around 2 million tonnes, with Norway, Chile and Scotland representing the three largest producers. While this industry has been highly successful in terms of expanding production and reaching new consumer markets, this has not been achieved without increasing the potential for environmental impact. The question of environmental impacts following the escape of farmed salmon, and in particular the potential for genetic interactions with wild conspecifics, continue to provide key themes for scientific debate and public controversy [1–3].

Thousands of farmed salmon are reported to escape from aquaculture installations on a regular basis and, due to the probability of underreporting [4–6], it has been estimated that the true number of escapees is likely to be significantly higher [7]. Depending upon several factors such as fish age and time of escapement [8, 9], some farmed salmon manage to survive in the wild and enter freshwater where native salmon populations reproduce. Farmed escapees have been observed on the spawning grounds of native populations in Norway [10, 11], the United Kingdom and Ireland [12–14], Iceland [15] Western Canada [16] and eastern North America [17]. While the reproductive success of farmed escapees is limited compared to wild salmon [18, 19], farmed salmon have been observed spawning in the wild [7, 12, 20], and genetic changes in native populations as a result of successful reproduction have been detected [21–24].

A recent study of historical and contemporary samples from 20 Norwegian salmon rivers estimated cumulative introgression of farmed escaped salmon in native populations [25]. Using a combination of single nucleotide polymorphisms (SNPs) and approximate Bayesian computation, these authors estimated introgression of farmed salmon reached nearly 50% in some rivers. This level of genetic introgression is of significant concern for two main reasons. First, wild Atlantic salmon populations are often genetically differentiated from one another and may be adapted to their specific rivers [26–29]. Thus, invasion of a non-local fish may disrupt local adaptation. Second, farmed Atlantic salmon have been subject to selection for a range of traits since breeding programs wer’s [30–32]. As a result, farmed salmon display a range of genetic differences to wild Atlantic salmon in a number of measured traits; for example, greatly increased growth rates under farming conditions [33–36], reduced predator awareness [37], reduced genetic diversity in highly polymorphic genetic markers at the population level [38, 39], and altered gene-transcription patterns [40, 41]. Furthermore, studies conducted in the wild have demonstrated that the offspring of farmed salmon display reduced survival compared to the offspring of wild salmon [19, 42–44], an observation consistent with the reported lower fitness of the offspring of hatchery fish in the wild [45, 46]. Studies of the genetic differences between wild and domesticated salmon therefore represent an important contribution towards gaining understanding of the likely evolutionary consequences of interbreeding between escaped salmon and their wild conspecifics.

Forty years ago King and Wilson proposed that gene regulation governs evolution of anatomy, physiology and behaviour [47, 48] and the development of broad-spectrum/high-throughput genomic approaches allows the theory to be tested. DNA microarrays, for example, are commonly used to simultaneously measure the mRNA expression levels of thousands of transcripts and have been available for salmonids since 2004 [49, 50]. As well as being employed to study genome-wide transcript expression, microarray experiments have been tailored to explore aspects of evolutionary processes, such as domestication in Atlantic salmon. In a series of microarray studies, Roberge and colleagues [40, 41] suggested that five to seven generations of selection for domestication may be sufficient to induce heritable alterations in transcription levels compared to wild populations. Of the differentially expressed genes that they detected, 16% displayed parallel changes among the strains, providing further evidence that artificial selection drives evolutionary changes at the gene transcription level [40]. Furthermore the authors suggested that, since most (82%) of the differentially expressed genes exhibited non-additive inheritance patterns, the consequences of introgression would likely to be difficult to predict [41].

In the present study, microarray analysis was used to explore potential gene transcription/regulatory consequences of hybridisation between wild and domesticated salmon. In order to investigate genome wide transcript expression differences between wild and domesticated stocks, mRNA levels were compared for yolk-sac and externally feeding fry originating from wild (Figgjo), domesticated (Mowi) and hybrid (Mowi ♀ x Figgjo ♂) populations reared under common conditions. Early life-history stages were focused upon, primarily to minimise transcriptional differences between the strains resulting solely from divergent inter-strain growth rates (up to three fold difference by four months post first feeding [36]). Furthermore, sampling during perceived periods of major physiological perturbation, *e.g.* hatching and swim up stages, were avoided, as individual variation during transition periods is likely to be critically influenced by sample timing. Body size differences in fish have been linked to developmental stage divergence and transcriptomic differences have been detected between size and age matched wild rainbow trout. Hence the exact methods employed to match life stages of wild and domesticated fish could influence the genes identified as differentially expressed between the stocks [51]. With the aim of minimising the confounding factors described above, this study was designed to provide an insight into genetic differences and interactions between wild and domesticated salmon, since understanding such interactions is essential both for the support of sustainable aquaculture practices and for the maintenance of healthy wild stocks.

## Methods

### Biological samples

The farmed salmon juveniles used for the present study originated from the Norwegian Mowi strain maintained by Marine Harvest at Tveitevåg, Norway. This represents one of the oldest commercial salmon strains, and at the time of stripping, the eggs and sperm used to generate the family-groups originated from approximately the 10^th^ generation. The Mowi strain was initially selected for increased growth, late maturation and high flesh quality through phenotypic selection, however, a family-based breeding program which included expansion in the numbers of traits being selected for was initiated in 1999 [34]. The Mowi strain has been demonstrated to display freshwater growth rates several times higher than various wild populations [34, 36, 52], and reduced survival compared to wild salmon under natural conditions when simultaneously planted out as eyed eggs [44].

The wild salmon used in this study originated from the Figgjo River in south west Norway. This population represents one of the most abundant in Norway, and is characterised by small to medium-sized fish (typically 1–2 sea winter returns). In the period 15-17^th^ October 2010, 24 wild fish were caught by rod and line angling in the river. These fish were transported to the local hatchery where they were held in tanks before being transported to the Matre research station in western Norway on 25^th^ October 2010. These fish were confirmed to be wild based upon scale growth patterns [53].

Both farmed and wild broodstock were stripped for gametes on 23^rd^ November 2010. A total of 30 families were created; 10 of each of the following crosses: pure wild, Figgjo ♀ × Figgjo ♂; hybrid, Mowi ♀ × Figgjo ♂; pure domesticated, Mowi ♀ × Mowi ♂. Fertilised eggs were placed into single family incubators and were held under standard hatchery conditions. At the eyed egg stage on 22^nd^ February 2011, families were pooled into duplicate experimental groups, *i.e*. six tanks in total, and by 23^rd^ March 2011 half of the eggs had hatched, these being termed 0°d post-hatch. The first sampling took place during fry yolk-sac re-absorption (256°d post-hatch) and then fish were transferred to heated (13°C) first feeding tanks. Fry were fed on standard hatchery diet (Skretting) 24 hr a day by automatic feeders according to a standard Skretting feeding table for appropriate temperatures. The second sampling took place 5 weeks into exogenous feeding (867°d post-hatch). The fish were starved for 24 hr prior to the second sample. For both sampling time points fry were euthanised with metacaine (Finquel^®^ Vet, Scanvacc, Årnes, Norway) overdose, with yolk sac fry being placed into RNALater^®^ (Life Technologies) and feeding fry being snap frozen on dry ice and stored at -70°C until homogenised.

The experiment was conducted in accordance with Norwegian regulations for the use of animals in research. No specific permits were required for this experiment because the fish were hatched and reared under standard aquaculture conditions without any form of experimental manipulation.

### Microarray experimental design

Microarray interrogations were performed using a custom-designed, oligonucleotide microarray platform (Agilent) with 44 K probes per slide (Salar_2; Agilent Design ID:025520). This microarray has been described in detail elsewhere [54] and further used/validated in a number of subsequent studies [55–57]. The design is logged with ArrayExpress (http://www.ebi.ac.uk/arrayexpress) under accession number A-MEXP-2065. Dual-label hybridisations were undertaken, with each experimental sample (Cy3 labelled) being competitively hybridised against a pooled reference control (Cy5 labelled) comprising equimolar amounts from each experimental RNA sample. The interrogations comprised 36 separate hybridisations; 3 states (wild × wild; farmed × wild, farmed × farmed) × 2 time-points (sac fry and fed fry) × 6 biological replicates. A single array was excluded from the analysis as it failed quality filtering, hence only five pools of domesticated feeding fry were analysed (Table 1).

**Table 1:** **A representation of the experimental design; each biological replicate comprising equal quantities of RNA from six individuals**

	Sac fry	Feeding fry
Wild; F ♀ × F ♂	6 pools	6 pools
Hybrid; M ♀ × F ♂	6 pools	6 pools
Domesticated; M ♀ × M ♂	6 pools	6 pools

### RNA extraction and purification

Whole fry (N = 216) were homogenised rapidly in 8 × volume Tri Reagent (Sigma–Aldrich^®^, St. Louis, U.S.A.) using a Polytron mechanical homogeniser (Kinematica PT 1300 D, Lucerne, Switzerland) and the RNA extracted following the manufacturer’s instructions. RNA quantity and quality were assessed by spectrophotometry (NanoDrop ND-1000, Thermo Scientific, Wilmington, U.S.A.) and agarose gel electrophoresis respectively. For each hybridisation sample, equal amounts of total RNA from six individuals were pooled, column-purified (RNeasy Mini Kit, Qiagen, Crawley, UK), and then re-quantified and quality assessed as described above.

### RNA amplification and labelling

Each pooled RNA sample was amplified (TargetAmp™ 1-Round Aminoallyl-aRNA Amplification Kit, Epicentre Technologies Corporation, Madison, Wisconsin, USA) according to the manufacturer’s instructions. Following quality control (Nanodrop quantification and agarose gel electrophoresis) each aRNA sample was indirectly labelled and purified. Briefly, Cy dye suspensions (Cy3 and Cy5) in sufficient quantity for all labelling reactions were prepared by adding 40 μL high purity dimethyl sulphoxide (Stratagene, Hogehilweg, The Netherlands) per tube of Cy dye (PA23001 or PA25001; GE HealthCare, Little Chalfont, Bucks, UK). Each sample (2.5 μg aRNA) was denatured at 75°C for 5 min and then 3 μL 0.5 M NaHCO_3_ pH8.5 and 1.5 μL Cy3 or 1.0 μL Cy5 dye was added achieving a total volume of 15 μL per reaction. Samples were incubated for an hour at 25°C in the dark, purified using Illustra AutoSeq G-50 Dye Terminator Removal Kit (Qiagen GE Healthcare) and concentration, dye incorporation and purity were assessed via spectrophotometer (NanoDrop) with products also visualised on a fluorescent scanner (Typhoon Trio, GE Healthcare).

### Microarray hybridisation and quality filtering

Hybridisation was performed over two consecutive days using the Agilent Gene Expression Hybridisation Kit (Agilent Technologies) as per manufacturer’s instructions. For each reaction, 825 ng Cy5 labelled reference pool and 825 ng Cy3 labelled individual samples were combined in 35 μL nuclease free water and then 20 μL fragmentation master mix added (consisting of 11 μL of 10X blocking agent, 2 μL 25× fragmentation buffer and 7 μL nuclease free water). The reactions were then incubated at 60°C in the dark for 30 mins, chilled on ice, and mixed with 57 μL 2× GEx Hybridisation buffer (pre heated to 37°C), Following centrifugation (18000 × g for 1 min) the samples were kept on ice until loaded (103 μL) in a semi randomised order onto the microarray slides. Samples from the six biological replicates were spread across different slides, Cy3 fluorescence content (dye incorporation rate × volume) was also taken into consideration. To aid scanning, samples with the most similar amounts of Cy3 were grouped on the same slide. Hybridisation was carried out in a rotating rack oven (Agilent Technologies) at 65°C, 10 rpm over 17 hours.

Following hybridisation, slides were subject to a number of washing steps performed in Easy-Dip™ slide staining containers (Canemco Inc., Quebec, Canada). First, each microarray and backing gasket was disassembled in Agilent Wash Buffer 1 and microarray slides were transferred to an Easy Dip rack submerged in Wash Buffer 1. Following 1 min incubation at room temperature (c. 20°C) and 150 rpm (Stuart Orbital Incubator), slides were briefly dipped into Wash Buffer 1 pre-heated to 31°C, then placed into Wash Buffer 2 (31°C) for 1 min at 150 rpm. Finally, the slides were transferred to acetonitrile for 10 s and then Agilent Stabilization and Drying Solution for 30 s. The slides were then air dried in the dark and scanned within two hours.

Scanning was carried out at 5 μm resolution on an Axon GenePix Pro scanner at 40% laser power. The “auto PMT” function was enabled to adjust PMT for each channel such that less than 0.1% of features were saturated and so that the mean intensity ratio of Cy3:Cy5 signal was close to one. Agilent Feature Extraction Software (v 9.5) was used to identify features and extract background subtracted raw intensity values that were then transferred to GeneSpring GX (v.12) software where the quality filtering and normalisation steps took place. Intensity values ≤ 1 were adjusted to 1 and a Lowess normalisation undertaken. Stringent quality filtering ensured that features that represented technical controls, saturated probes, probe population outliers or probes which were not significantly different from the background were removed. Agilent feature extractions software was used to determine whether a probe was positive and significant based on a 2-sided *t*-test, indicating if the mean signal of a feature is greater than the corresponding background. A probe was retained if it was positive and significant in at least 75% of the arrays in any 2 of the experimental groups. This left 33,688 of the original 43,466 probes available for downstream analysis. A single array was excluded from the analysis as it was flagged as sub-standard by the feature extraction software and also appeared as a clear outlier on a Principal Component Analysis performed within Genespring in order to compare arrays. Thus 35 of the 36 arrays were statistically analysed.

Details of microarray experiment have been submitted to ArrayExpress under accession number E-MTAB-2578. The recording of the microarray experimental metadata complies with Minimum Information About a Microarray Experiment (MIAME) guidelines.

### Microarray data analysis

**Figure 1:**
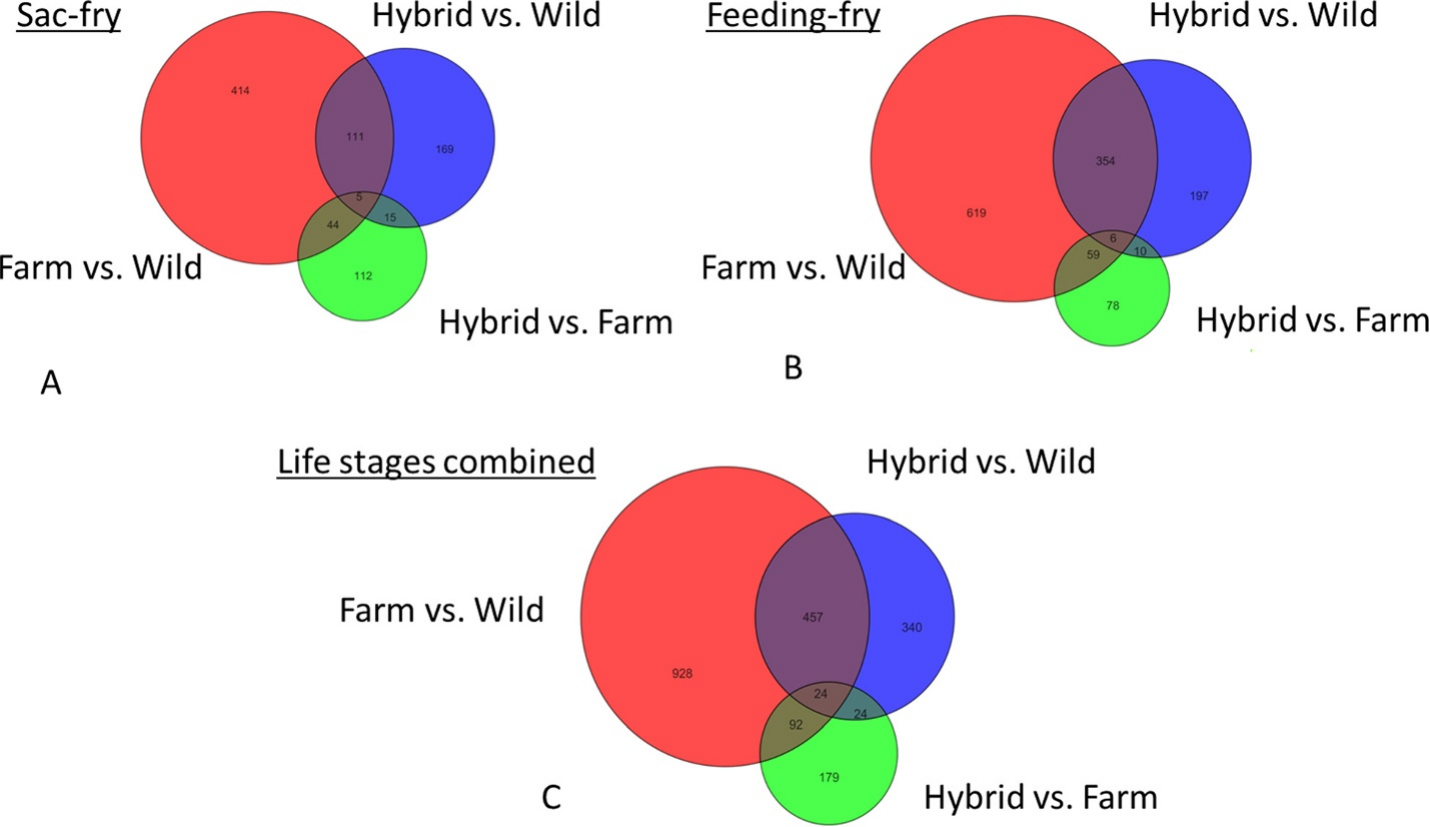
**A comparison of the number of differentially expressed transcripts between groups and life stages, based on T-tests (unpaired unequal variance) without multiple testing correction, p ≤ 0.01 and fold-change cut off at 1.3.** Panel **A** and **B** represent differences detected in the sac and feeding fry stage respectively, whereas panel **C** shows combined the differences; i.e.: each comparison is the union of the differences detected in the life stages.

**Figure 2:**
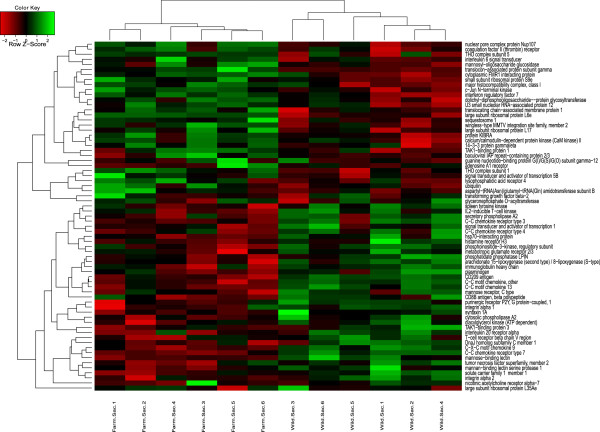
**Hierarchical clustering based on normalised intensity values of the essential genes of the significant pathways detected in sac fry.**

**Figure 3:**
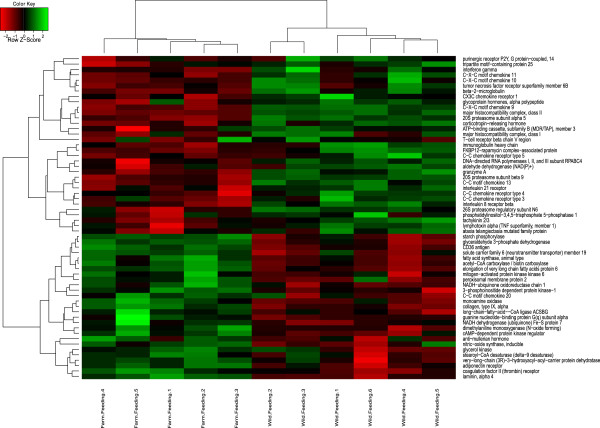
**Hierarchical clustering based on normalised intensity values of the essential genes of the significant pathways detected in feeding fry.**

**Figure 4:**
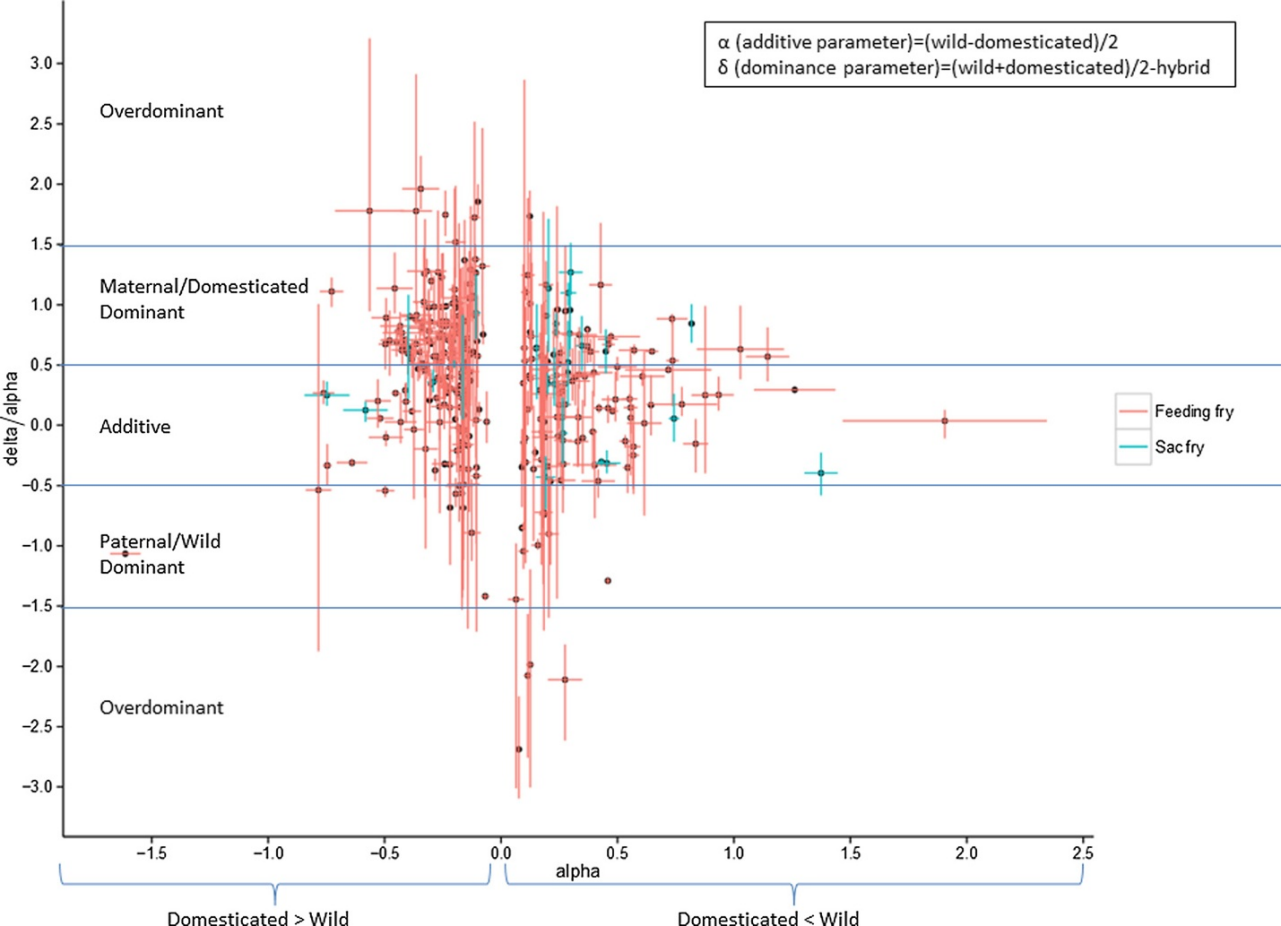
**Visual representation of heritability of annotated transcripts differentially expressed between experimental groups based on 1-way ANOVA (10% FDR).** Error bars show the standard deviation between replicate arrays. Nine overdominant, one dominant and one recessive transcript were excluded from the graph for easier visualisation.

Differentially expressed genes between the crosses were identified in GeneSpring using a number of statistical methods and criteria. For the entire data analysis, life stages were treated separately and to identify differentially expressed genes between experimental groups pairwise T-tests (unpaired unequal variance, p ≤ 0.01) were performed and a minimum fold change of 1.3 applied. These lists formed the basis of the Venn diagram (Figure 1). In contrast, the functional analysis of the genetic differences between wild and domesticated fish was based on less stringent criteria, with a p ≤ 0.05 and with no fold change requirement and were further analysed in R v.3.0.2 (R Core Team, 2014). This enabled sufficient KEGG annotation for the pathway analysis which in turn narrowed the list of unique genes by further filtering on significant pathways using the gage function of the GAGE package (Generally Applicable Gene-set/Pathway Analysis) [55], q ≤ 0.1) thereby increasing confidence despite the lenient initial comparison. The significant pathways (Table 2) were further analysed using the esset.grp and essGene functions [55] to identify non-redundant pathways and genes that changed over and above the noise level (Figures 2 and 3) respectively. Since pathways belonging to the human disease functional group are difficult to interpret in fish, this group was excluded from the gene enrichment analysis. Genes that were involved in any of the significantly perturbed pathways and changed beyond one standard deviation from the mean of all genes were subject to hierarchical clustering (Pearson correlation) and are presented on the heat maps using gplots package [56]. To look at heritability of differentially expressed genes between stocks, 1-way ANOVA (unequal variance) was performed with 10% FDR (Benjamini-Hochberg). To avoid repeat counting of the same gene, only transcripts that had BLASTx and/or KEGG annotation were chosen and where multiple probes were present for the same gene, the probe with the highest significance was chosen. For the unique genes obtained, additivity; α = (wild-domesticated)/2 and dominance parameters; δ = (wild + domesticated)/2-hybrid were calculated from normalised intensity values and α and δ/α were plotted using the ggplot2 package (Figure 4) [57].

**Table 2:** **Significantly differentially represented KEGG pathways (multiple testing corrected p ≤ 0.1) between wild and domesticated stocks in the two life stages, wild fish is considered as control**

	***KEGG functional group***	***KEGG sub-group***	***KEGG pathway***	***Direction of perturbation***	***p-value***	***Set size***
Sac fry	**Cellular Processes**	**Cell growth and death**	**Oocyte meiosis**	**Up regulated**	**0.00212**	**15**
	**Environmental Information Processing**	**Signal transduction**	**Hippo signaling pathway**	**Up regulated**	**0.00128**	**15**
	**Environmental Information Processing**	**Signal transduction**	**Wnt signaling pathway**	**Up regulated**	**0.00053**	**20**
	**Genetic Information Processing**	**Folding, sorting and degradation**	**Protein processing in endoplasmic reticulum**	**Up regulated**	**0.00186**	**36**
	**Genetic Information Processing**	**Translation**	**Aminoacyl-tRNA biosynthesis**	**Up regulated**	**0.00734**	**13**
	**Genetic Information Processing**	**Translation**	**Ribosome**	**Up regulated**	**0.00016**	**50**
	**Genetic Information Processing**	**Translation**	**Ribosome biogenesis in eukaryotes**	**Up regulated**	**0.00024**	**31**
	**Genetic Information Processing**	**Translation**	**RNA transport**	**Up regulated**	**0.00002**	**39**
	**Cellular Processes**	**Transport and catabolism**	**Phagosome**	**Down regulated**	**0.00042**	**37**
	**Environmental Information Processing**	**Signal transduction**	**NF-kappa B signaling pathway**	**Down regulated**	**0.00093**	**25**
	**Environmental Information Processing**	**Signaling molecules and interaction**	**Cytokine-cytokine receptor interaction**	**Down regulated**	**≤0.00001**	**26**
	**Organismal Systems**	**Immune system**	**B cell receptor signaling pathway**	**Down regulated**	**0.00133**	**16**
	**Organismal Systems**	**Immune system**	**Chemokine signaling pathway**	**Down regulated**	**≤0.00001**	**38**
	**Organismal Systems**	**Immune system**	**Complement and coagulation cascades**	**Down regulated**	**0.00385**	**21**
	Organismal Systems	Immune system	Fc epsilon RI signaling pathway	Down regulated	0.00778	16
	**Organismal Systems**	**Immune system**	**Hematopoietic cell lineage**	**Down regulated**	**0.00007**	**14**
	**Organismal Systems**	**Nervous system**	**Glutamatergic synapse**	**Down regulated**	**0.00152**	**19**
	**Organismal Systems**	**Nervous system**	**Serotonergic synapse**	**Down regulated**	**0.00303**	**16**
	**Organismal Systems**	**Nervous system**	**Synaptic vesicle cycle**	**Down regulated**	**0.00171**	**18**
	**Metabolism**	**Lipid metabolism**	**Glycerophospholipid metabolism**	**Down regulated**	**0.00781**	**10**
	**Environmental Information Processing**	**Signal transduction**	**NF-kappa B signaling pathway**	**Two way perturbed**	**0.00116**	**25**
	Environmental Information Processing	Signal transduction	TNF signaling pathway	Two way perturbed	0.00368	19
	**Environmental Information Processing**	**Signaling molecules and interaction**	**Cytokine-cytokine receptor interaction**	**Two way perturbed**	**0.00001**	**26**
	**Environmental Information Processing**	**Signaling molecules and interaction**	**Neuroactive ligand-receptor interaction**	**Two way perturbed**	**0.00371**	**23**
	**Organismal Systems**	**Development**	**Osteoclast differentiation**	**Two way perturbed**	**0.00555**	**28**
	**Organismal Systems**	**Immune system**	**Chemokine signaling pathway**	**Two way perturbed**	**0.00343**	**38**
	Organismal Systems	Immune system	NOD-like receptor signaling pathway	Two way perturbed	0.00416	14
	**Organismal Systems**	**Immune system**	**Toll-like receptor signaling pathway**	**Two way perturbed**	**0.00240**	**19**
	**Metabolism**	**Lipid metabolism**	**Glycerophospholipid metabolism**	**Two way perturbed**	**0.00275**	**10**
Feeding fry	**Cellular Processes**	**Transport and catabolism**	**Peroxisome**	**Up regulated**	**0.00014**	**27**
	**Environmental Information Processing**	**Signaling molecules and interaction**	**ECM-receptor interaction**	**Up regulated**	**0.01210**	**12**
	Organismal Systems	Circulatory system	Cardiac muscle contraction	Up regulated	0.00939	20
	**Organismal Systems**	**Digestive system**	**Fat digestion and absorption**	**Up regulated**	**0.00047**	**16**
	**Organismal Systems**	**Digestive system**	**Pancreatic secretion**	**Up regulated**	**0.00062**	**17**
	**Organismal Systems**	**Digestive system**	**Protein digestion and absorption**	**Up regulated**	**0.00004**	**23**
	Organismal Systems	Endocrine system	Adipocytokine signaling pathway	Up regulated	0.00435	15
	**Organismal Systems**	**Endocrine system**	**Insulin signaling pathway**	**Up regulated**	**≤0.00001**	**19**
	**Organismal Systems**	**Endocrine system**	**PPAR signaling pathway**	**Up regulated**	**≤0.00001**	**29**
	**Metabolism**	**Amino acid metabolism**	**Arginine and proline metabolism**	**Up regulated**	**0.00014**	**17**
	**Metabolism**	**Amino acid metabolism**	**Glycine, serine and threonine metabolism**	**Up regulated**	**0.00029**	**17**
	**Metabolism**	**Carbohydrate metabolism**	**Glycolysis/Gluconeogenesis**	**Up regulated**	**0.00001**	**25**
	**Metabolism**	**Carbohydrate metabolism**	**Propanoate metabolism**	**Up regulated**	**0.00084**	**12**
	Metabolism	Energy metabolism	Carbon fixation in photosynthetic organisms	Up regulated	0.00091	12
	**Metabolism**	**Energy metabolism**	**Methane metabolism**	**Up regulated**	**0.00048**	**12**
	**Metabolism**	**Energy metabolism**	**Oxidative phosphorylation**	**Up regulated**	**0.00058**	**59**
	**Metabolism**	**Lipid metabolism**	**Biosynthesis of unsaturated fatty acids**	**Up regulated**	**0.00004**	**12**
	Metabolism	Lipid metabolism	Fatty acid degradation	Up regulated	0.00056	16
	Metabolism	Lipid metabolism	Fatty acid elongation	Up regulated	0.00742	11
	Metabolism	Lipid metabolism	Glycerolipid metabolism	Up regulated	0.00429	15
	Environmental Information Processing	Signal transduction	Jak-STAT signaling pathway	Down Regulated	0.00005	16
	**Environmental Information Processing**	**Signal transduction**	**NF-kappa B signaling pathway**	**Down Regulated**	**≤0.00001**	**28**
	**Environmental Information Processing**	**Signaling molecules and interaction**	**Cytokine-cytokine receptor interaction**	**Down Regulated**	**≤0.00001**	**38**
	**Environmental Information Processing**	**Signaling molecules and interaction**	**Neuroactive ligand-receptor interaction**	**Down Regulated**	**0.00122**	**22**
	**Organismal Systems**	**Immune system**	**Antigen processing and presentation**	**Down Regulated**	**0.00267**	**22**
	Organismal Systems	Immune system	Chemokine signaling pathway	Down Regulated	≤0.00001	35
	**Organismal Systems**	**Immune system**	**Cytosolic DNA-sensing pathway**	**Down Regulated**	**0.00799**	**10**
	**Organismal Systems**	**Immune system**	**Fc epsilon RI signaling pathway**	**Down Regulated**	**0.00130**	**12**
	Organismal Systems	Immune system	Fc gamma R-mediated phagocytosis	Down Regulated	0.00407	17
	Organismal Systems	Immune system	Toll-like receptor signaling pathway	Down Regulated	0.00055	17
	**Genetic Information Processing**	**Folding, sorting and degradation**	**Proteasome**	**Down Regulated**	**≤0.00001**	**25**
	**Environmental Information Processing**	**Signaling molecules and interaction**	**Cytokine-cytokine receptor interaction**	**Two way perturbed**	**≤0.00001**	**38**

Set size is the number of genes included in the gene set test. Non-redundant pathways are shown in bold.

### RT-qPCR validation

Expression of five selected genes was validated using real time quantitative polymerase chain reaction (RT-qPCR). Genes of interest were chosen based on their p-values in either of the life stages and/or fold changes across experimental groups. Two additional ‘housekeeping’ genes were included in the analysis for normalisation purposes. Reference genes were selected from the literature or based on their constant/steady expression profile in the microarray analysis. Details of the selection criteria, primer design and RT-qPCR are given in (Additional file 1).

cDNA was synthesised from 1 μg of column-purified total RNA per sample using the High-Capacity cDNA RT kit (Applied Biosystems, Paisley, U.K.), following manufacturer’s instructions, but using a mixture of the random primers (1.5 μL as supplied) and anchored oligo-dT (0.5 μL at 400 ng/μl). Negative controls lacking reverse transcriptase were included to check for genomic DNA contamination. A pool comprising similar amounts of all cDNA samples was used in a dilution series to determine primer efficiencies. The remaining cDNAs were then diluted 20-fold in water.

qPCR amplifications were carried out in duplicate 20 μL reaction volumes, containing either 5 μL of cDNA (1/20 dilution) or no enzyme control (1/20 dilution) or serially-diluted cDNA pools (ranging from 1/10 to 1/640 dilution) or water (no template control) and 0.5 μM each primer and 10 μL ABgene Sybr Green (2×; Thermo Scientific, Wilmington, U.S.A.). All qPCR reactions were performed using the following thermal profile: initial activation at 95°C for 15 min, amplification through 40 cycles of 95°C for 15 s, 60°C for 15 s and 72°C for 30 s. Following the amplification phase, a melt curve analysis was performed to confirm the amplification of a single product. In addition, to determine the size and identity of the amplicons, agarose gel electrophoresis of amplicons was undertaken. Data were analysed in REST 2009 software [58].

## Results and discussion

### Differentially expressed transcripts between strains and life stages

For the purposes of statistical analysis, life stages were treated separately. In order to identify differentially expressed genes between experimental groups, pairwise T-tests (unpaired unequal variance, p ≤ 0.01, fold change ≥ 1.3) were used. The largest differences in transcription were observed between the domesticated and wild groups, however, it is interesting to note that there were fewer significantly differentially expressed transcripts between fish of hybrid and domesticated origin (176 in sac fry and 153 in feeding fry), than between wild and hybrids (300 and 567 respectively) (Figure 1A and 1B). Maternal effects might have contributed to the bias, as hybrid eggs were originated from domesticated females. In addition to direct genetic effects from the yolk sac, such as highly abundant maternal ribosomes and maternally deposited RNAs, other yolk sac components, such as hormones, proteins or nutrients can influence the offspring’s genomic activity by modifying or interacting with its transcription factors or DNA structure [59]. It was also noteworthy that there were over 1.8 times as many differentially expressed entities detected in the exogenous feeding stage than in the yolk-sac samples in the wild-domesticated and hybrid-wild comparisons (Figure 1). The initiation of exogenous feeding is known to alter gene expression through the activation of certain metabolic pathways, such as the glycolytic pathway enabling the utilisation of exogenous feeds or fatty acid pathways facilitating lipid metabolism and deposition [60]. This was reflected in the observation that differentially expressed genes belonging to carbohydrate and lipid metabolism pathways were common in feeding fry, but not in sac fry. Furthermore, the hatchery diet employed, containing plant derivatives and thus poorly matching the usual diet of wild fish, might affect gene expression differentially in wild and domesticated stocks, and may thereby account for some of the differences detected in the feeding stage. However, the initiation of exogenous feeding did not increase the number of differentially expressed transcripts between domesticated fish and their hybrids, despite the expected fading of maternal effects in later life stages [59]. Although some of the significantly differentially expressed genes overlapped between life stages, sampling at two time points revealed a number of life stage specific patterns (Figure 1C).

### Functional classification of differentially expressed genes between wild and domesticated strains

It is difficult to make comparisons between studies at the level of differentially expressed genes due to the use of different stocks, life stages, tissues and microarray designs. Although common genes are rarely reported, biological pathways and even more so functional classes tend to overlap between studies [40]. To characterise the functional significance of the transcripts that were differentially expressed between wild and domesticated fish, we assigned KEGG annotations to them, unique genes were then subject to gene enrichment analysis (Table 2).

Transcriptional changes between wild and domesticated fish varied according to functional group life stage considered (Table 2). Among the differentially expressed transcripts, the ones relating to the immune system were significantly over-represented in both life stages. In addition, disproportionately large numbers of differentially expressed transcripts were detected for the nervous and digestive systems in sac fry and feeding fry respectively (Table 2). An interesting parallel to this trend has been reported from transcriptomic comparisons between normal and dwarf lake white fish (*Coregonus* spp.), where the authors stressed the importance of survival functions in dwarf individuals and growth related functions in normal fish [61]. The majority of differentially expressed immune related transcripts were down-regulated in domesticated animals, whereas the opposite was observed for transcripts associated with the digestive system (Table 2). Such apparent trade-offs between growth and immune response have also been documented in Atlantic salmon by previous authors [52, 62]. It has been suggested that selection for growth could therefore favour individuals with more active endocrine regulatory components [63] and this is supported by the findings that most differentially expressed transcripts relating to the digestive system showed higher expression in domesticated individuals as did endocrine system related transcripts (Table 2). In contrast, transcripts with nervous system and environmental information processing roles were mainly down-regulated in the domesticated strain, which might be explained by the relatively homogeneous and controlled environment experienced by domesticated individuals. Tymchuk and colleagues reported a down regulation of cell division in the brain of domestic rainbow trout, despite conducting their experiment on size-matched fish [64]. The relationship of wild : domesticated transcripts involved in energy metabolism, protein synthesis, stress and immune response, response to stimuli and digestion are in agreement between this study and previous studies investigating effects of domestication in salmonids [40, 41, 51, 65]. Dishevelled Segment Polarity Protein 2 (DVL2), a member of the Wnt signalling pathway, was hypothesised in previous work to show footprints of selection through domestication in Atlantic salmon [66]. Although oligo probes for this particular gene were not incorporated in the design of *Salar_2*, the Wnt signalling pathway was significantly up regulated in the sac fry stage.

A number of differentially expressed pathways were common between life stages, further increasing confidence in their significance. Toll-like receptor interaction, NF-kappa B signalling and cytokine-cytokine interactions pathways were down-regulated in the domesticated strain at both sampling points (Table 2). Toll-like receptors are primary sensors detecting a wide variety of microbial components and triggering innate immune responses through activating the transcription factor nuclear factor-kappaB, which controls the expression of inflammatory cytokine genes [67]. Cytokines have the ability to regulate endocrine activity and stress hormones and, in addition to immune activation they are likely to play a role in a number of interrelated processes, such as food intake efficiency, energy balance and tissue metabolism [68], and could thus provide a linking element between the differentially expressed pathways identified in this study.

To visualise expression patterns of the key genes belonging to identified significant pathways, hierarchical clustering was performed and expression intensities are shown on heat maps for the two life stages (Figures 2 and 3). Although universal transcript-level differences have not been identified when studying different wild and domesticated strains, there are a small number of genes that have been reported to be differentially expressed by more than one study. Parallel changes included ATP synthase, growth hormone receptor [39], cytochrome this study, [39, 52, 65], solute carrier family members (this study [51, 65]), glyceraldehyde 3-phosphate dehydrogenase this study, [39] and malate/NADH dehydrogenase this study, [39, 65]. A number of immune related transcripts such as lectin and various CD and MHC family members were also reported by multiple sources, however their direction of change varies between studies this study, [39, 52, 64, 65]. This might be due to the high specificity of the pathogen induced chemokine regulation [69].

### Heritability predictions of differentially expressed genes

To shed light on the inheritance patterns of the genes differentially expressed between stocks gene expression additivity was studied. 1-way ANOVAs were performed with multiple testing corrections (corrected p ≤ 0.1) and only unique genes (see Methods for details); 25 in sac fry and 313 in feeding fry were included in the analysis (Additional file 2). By calculating the ratio of the dominance parameter, δ = (wild + domesticated)/2-hybrid and the additive parameter, α = (wild-domesticated)/2 one can estimate the inheritance pattern of genes from their expression values. By definition a transcript whose hybrid gene expression value corresponds to the mid value of the parents is additive, whereas a transcript whose hybrid gene expression value resembles more closely one parent or another is dominant. δ/α = 0 corresponds to a state of perfect within-locus additivity (i.e.; δ = 0) and δ/α = 1 or -1 corresponds to complete dominance. According to logic and an assumption used by Renaut *et al*. (2009) in halving the intervals, we can presume that transcripts resemble:

- Additivity if -0.5 < δ/α < 0.5
- Paternal/Wild dominance if -1.5 < δ/α < -0.5
- Maternal/Domesticated dominance if 0.5 < δ/α < 1.5
- Overdominance if δ/α falls out of the interval -1.5-1.5

According to our results (Figure 4), most transcripts found to be differentially expressed between stocks showed either additive; 48% and 45% or maternal/domesticated dominant; 52% and 42.2% heritance patterns in sac fry and feeding fry respectively. In addition, 6.1% of transcripts were paternal dominant and 6.7% were overdominant in the feeding stage. Among the overdominant transcripts, the ones considered to be more similar to the mother’s expression were approximately three times more abundant than the ones found to be closer to the father’s. Additivity, as an important mode of inheritance between diverged intraspecific populations, has been reported in previous gene expression studies conducted on wild and domesticated salmon [65] and brook charr [70] as well as on dwarf and normal lake white fish [71]. Additive genetic variation was also found to influence a number of traits in Atlantic salmon such as fitness, survival [3, 72], growth and behaviour [34, 36, 72]. In addition to additivity, the findings of this study are indicative of the relevance of a dominant inheritance pattern in wild-domesticated hybrids. However, since the hybrids in this study were produced only by crossing a domesticated dam with a wild sire, we are unable to conclude whether the dominance is purely caused by maternal effects or if the domesticated strain has a superior influence on the transcription of the offspring too. The importance of maternal dominance was highlighted by Bougas and colleagues when studying the transcriptional landscape of wild and domesticated brook charr hybrids. Similarly to the results reported here, their comparison of domesticated and anadromous wild fish revealed that 54.3% of the differentially expressed transcripts exhibited an additive inheritance pattern, 40% showed maternal, 5% paternal dominance, and a small number of transcripts were over/under dominant [70]. Contrary to the current findings, Debes *et al*. reported that 26.8% of the wild-domesticated Atlantic salmon hybrid transcripts showed wild dominance [65]. There are a number of variables between the experiments that might account for the differences observed between the studies. First, since different tissue types (gill vs whole fry) were used in the studies, tissue specific gene expression might have affected the results. Second it is likely that the different parental strains crossed had different genetic architecture, which could have affected the gene expression of the offspring. In addition, Debes *et al*. report the use of reciprocal hybrids, whereas in this study, hybrid eggs originated only from domesticated animals. Third, since parental effects vary over time, and seem to be most pronounced at the yolk sac resorption stage, and tend to decrease over time, the sampling time-point selected could also have contributed to the gene expression differences of the hybrids [73]. Indeed, in the current study a higher proportion of genes showed a dominant inheritance pattern at the yolk sac stage (52%) then during exogenous feeding (42%), suggesting stronger maternal influence at the earlier life stage. Tissue specificity, the time spent under selection pressure and the genetic architecture of the parental strains might have contributed to the disagreement between our results and a study reporting equal additive, recessive and dominant regulation when analysing the heritability of transcription in livers of wild and domesticated rainbow trout [51].

### RT-qPCR validation of the results

Four significantly differentially regulated transcripts were chosen for further investigation via RT-qPCR, based on their p-values and fold changes. In addition, IGF-1 was also included in the RT-qPCR experiment due to its hypothesised functional importance in the process of domestication [35] and despite the fact that no significant gene expression difference was detected for this transcript on the microarray. Although fold changes were generally low, a good correspondence of expression ratio and direction of regulation was obtained between the microarray and RT-qPCR for most genes quantified (Table 3). Consistent with the microarray data, RT-qPCR results also showed no significant difference in expression of IGF-1 between experimental groups. In contrast, Solberg et al. found elevated IGF-1 mRNA levels in domesticated and hybrid Atlantic salmon head kidneys compared to those of wild fish [35]. The disagreement between our results might be due to the different strains, life stages and tissue types (head kidney vs whole fry) used in the studies.

**Table 3:** **A comparison of gene expression ratios of domesticated and hybrid salmon with respect to wild individuals evaluated using RT-qPCR and microarray analysis**

	Sac fry				Feeding fry			
	Domesticated		Hybrid		Domesticated		Hybrid	
**Target**	**RT-qPCR**	**MA**	**RT-qPCR**	**MA**	**RT-qPCR**	**MA**	**RT-qPCR**	**MA**
MHCII	-1.48	-1.37	(-1.17)	(-1.10)	-1.95	-2.09	-1.24	-1.38
EPHX	1.27	2.24	1.20	1.57	1.23	2.08	1.20	1.55
IGF	-1.11	(1.39)	(1.01)	(1.56)	(1.08)	(-1.14)	(1.05)	(1.79)
Pesc	(1.02)	2.82	(1.03)	1.91	-1.15	2.43	-1.10	(1.36)
Poly10	-2.31	-6.72	-1.28	(-1.78)	-1.61	-3.19	-1.28	-1.63

Microarray values are based on T-tests (unpaired unequal variance, p ≤ 0.01 and FC > =1.3), whereas RT-qPCR ratios were obtained by REST2009 (p ≤ 0.05). Non-significant values are shown in parenthesis. Ratios lower than 1 are expressed as -1/ratio to obtain an equivalent value to ratios above 1.

## Conclusions

This study investigated transcriptional differences between wild and domesticated Atlantic salmon at the early life-history stages, before developmental/growth rate between them could substantially influence experimental outcome. According to the results of this study, genetic information processing and translation pathways in particular are up regulated in domesticated fish whereas immune system related pathways are down regulated in the yolk sac stage. During early exogenous feeding, the digestive and endocrine systems as well as carbohydrate, energy and lipid metabolism pathways are more highly expressed in the domesticated strain, while environmental information processing and immune pathways, especially those related to cytokines, are suppressed compared to those of wild stock.

While sampling complications following growth divergence between stocks need to be considered, it is important to study different life-stages to explore developmental state-specific differences between wild and domesticated individuals and the possible influence of common rearing on gene expression (*i.e*. translocation of wild fish into a hatchery environment). This study re-enforces the necessity of studying reciprocal hybrids in order to differentiate between maternal (and potentially epigenetic) and domestication effects influencing heritability. Finally, these data support the view that the effect of introgression is highly dependent on the population specific genetic architectures of the crosses [41, 51, 74], thus studies conducted on multiple strains are essential to draw general conclusions regarding the outcome of genetic interactions between wild and farmed fish.

### Availability of supporting data

Details of microarray experiment have been submitted to ArrayExpress under accession number E-MTAB-2578 and are accessible at http://www.ebi.ac.uk/arrayexpress/experiments/E-MTAB-2578/. The recording of the microarray experimental metadata complies with Minimum Information About a Microarray Experiment (MIAME) guidelines.

## Electronic supplementary material

Additional file 1: **Details of the RT-qPCR validation.** Data consists of sequence information for the RT-qPCR primers, the results of the RT-qPCR, including the output of REST and a comparison of the microarray and RT-qPCR results. (XLSX 227 KB)

Additional file 2: **Heritability data.** The list of genes the heritability scatter graph is based on, including their significance values, normalized intensity values and annotations. (XLSX 25 KB)
